# Phase II trail of nab-paclitaxel in metastatic breast cancer patients with visceral metastases

**DOI:** 10.1186/s12885-021-08921-2

**Published:** 2021-11-02

**Authors:** Yizhao Xie, Chengcheng Gong, Jian Zhang, Leiping Wang, Jun Cao, Zhonghua Tao, Ting Li, Yannan Zhao, Yi Li, Shihui Hu, Biyun Wang, Xichun Hu

**Affiliations:** 1 Department of Medical Oncology, Fudan University Shanghai Cancer Center, No.270, Dong’an Road, Xuhui District, Shanghai, 200032 China; 2 Department of Oncology, Shanghai Medical College, Fudan University, Shanghai, China

**Keywords:** Metastatic breast Cancer, Chemotherapy, Nab-paclitaxel

## Abstract

**Background:**

Visceral metastases account for 48–67% of metastatic breast cancer (MBC) patients and presage a worse overall survival. Previous study suggested potential effect of nab-paclitaxel on patients with visceral metastases subgroups. This phase II trial was conducted to explore the efficacy and safety of nab-paclitaxel in such a high-risk group of patients.

**Methods:**

In this prospective, single-center, open-label, phase II study, MBC patients with visceral metastases (*N* = 80) received nab-paclitaxel (Abraxane, 125 mg/m2, D1, D8, D15 every 28 days).

**Results:**

The median PFS was 5.1 months (95% CI: 4.2–6.0 months), with an ORR of 33.8% (95% CI 21.3–43.8%) and CBR of 66.2% (95% CI 56.3–75.0%). In univariate analysis, patients with premenopausal status had a trend of better treatment outcome. Multivariate analysis demonstrated non brain metastasis (adjusted HR 0.31, 95% CI 0.12–0.83, *P* = 0.019) and first line treatment (adjusted HR 0.37, 95% CI 0.17–0.81, *P* = 0.013) as independent predictors of longer PFS. The overall safety was acceptable with most common treatment-related, grade ≥ 3 toxicities of neutropenia (16.3%) and sensory neuropathy (3.7%).

**Conclusions:**

This phase II trial documented satisfactory efficacy and safety of nab-paclitaxel in MBC patients with visceral metastases, providing evidence for relative clinical practice. Patients in first line therapy had better treatment outcome. For patients with premenopausal status or brain metastasis, further alternatives (for example, combined chemotherapy or targeting therapy) might be required. This study also demonstrated the efficacy and safety of 125 mg/m2 nab-paclitaxel among Asian patients.

**Trial registration:**

This research is registered under clinicaltrials.gov (NCT 02687490, February 22, 2016).

## Introduction

Among female malignancy, breast cancer (BC) remains the most common type and a primary cause of cancer-related death worldwide, leading to nearly 464 thousand deaths per year, mostly for metastatic breast cancer (MBC) [1, 2]. The incidence and mortality of breast cancer keep growing in China as well, according to epidemiologic study [3].

Visceral metastases were found in 48–67% patients with metastatic breast cancer [4, 5]. Studies showed that patients with visceral metastases had worse treatment outcome and shorter overall survival (OS) compared to other MBC patients, suggesting that visceral metastasis was a highly poor prognostic factor in terms of breast cancer [4–7]. Furthermore, National Comprehensive Cancer Network (NCCN) guideline recommends chemotherapy to luminal type MBC with visceral crisis [8].

Nanoparticle albumin-bound (nab)-paclitaxel is a biologically interactive, albumin-bound formation of paclitaxel particle developed to avoid or minimize the toxicities associated with traditional paclitaxel and docetaxel such as sensory neuropathy, neutropenia and severe hypersensitivity. Preclinical study showed that nab-paclitaxel had 33% higher paclitaxel concentration to tumors and enhanced transport across endothelial cells compared to standard paclitaxel [9]. A phase III trial enrolled 454 MBC patients and randomized them into 3-week cycles of either 260 mg/m2 nab-paclitaxel or 175 mg/m2 standard paclitaxel, showing results of a significantly better overall response rate (ORR, 33% vs 19%; *P* = 0.001), longer time to progression (TTP, 5.3 vs 3.9 months; *P* = 0.006) and subgroup analysis showed a significant higher ORR in visceral disease patients of nab-paclitaxel group compared to standard group (34% vs 19%, *p* = 0.002), [10]. Another phase two trial explored three different nab-paclitaxel doses (300 mg/m2 q3w, 100 mg/m2, or 150 mg/m2 qw) and docetaxel 100 mg/m2 q3w for untreated patients with MBC and indicated that the 150 mg/m2 qw regimen of nab-paclitaxel showed extended progression free survival (PFS) than docetaxel (12.9 vs 7.5 months, *P* = 0.0065), with lower rate of grade 3/4 neutropenia, febrile neutropenia, and fatigue, moreover, 84% patients in this trial had visceral metastases [11]. After permission by the drug administration, real world data also indicated safety and efficacy of nab-paclitaxel in clinical use [12, 13].

Since subgroup analysis indicated a potential positive effect of nab-paclitaxel in visceral metastasis patients, here we conducted a phase II clinical trial of nab-paclitaxel in MBC patients with visceral metastases. The present study aimed to explore the efficacy and safety of nab-paclitaxel in such a high-risk group of patients. We also explored the use of 125 mg/m2 nab-paclitaxel among Asian patients.

## Methods

This prospective, single-center, open-label, phase II study was conducted in Fudan University Shanghai Cancer Center. The protocol and related materials were approved by the appropriate institutional review boards and independent ethics committees. All process of this study was incompliance with the Declaration of Helsinki and the relevant guidelines. All of the patients signed written informed consent forms before study. This research is registered under clinicaltrials.gov (NCT 02687490).

### Patients

Eligible patients were nonpregnant, nonlactating 18 to 70 years old females with histologically or cytologically confirmed, measurable MBC with an expected survival of more than 12 weeks. Patients were included if they had radiologically or histologically confirmed visceral dominant metastases; were expected to acquire benefit from chemotherapy; received paclitaxel in metastatic setting should be proven effective to prior paclitaxel based regimen and disease progressed after at least 3 months from the last administration of paclitaxel; received paclitaxel as neoadjuvant/adjuvant therapy can be enrolled if disease relapsed after at least 6 months from the completion of neoadjuvant/adjuvant chemotherapy; had acceptable clinical laboratory test results at baseline.

Patients were excluded from participation if they had an Eastern Cooperative Oncology Group performance (ECOG) status of more than 2; received treatment with other experimental drug within 4 weeks before enrollment; received radiotherapy of axial bones within 4 weeks before enrollment or lack of recovery from prior radiotherapy; had symptomatic central nervous system metastases; had uncontrolled serious infection; or had other malignancy within the previous 5 years except nonmelanoma skin cancer, cervical intraepithelial neoplasia, or in situ cervical cancer.

### Treatments

Patients were treated with nab-paclitaxel (Abraxane, 125 mg/m2, D1, D8, D15 every 28 days) until disease progression (PD), death, unacceptable toxicity, or physician’s decision. Combined treatments were allowed based on physician’s choice, for instance, trastuzumab and bevacizumab.

### Assessments

All patients included in the study were evaluated with appropriate cross sectional imaging studies for disease response at baseline and every 8 weeks. Complete response (CR), partial response (PR), stable disease (SD) and PD were defined and assessed according to the Response Evaluation Criteria in Solid Tumors (RECIST) 1.1. CRs and PRs required subsequent confirmation of response at least 4 weeks later.

The primary efficacy measure was PFS; secondary efficacy measures were ORR, OS and safety. PFS was defined as the time from study registration to disease progression or death from any cause. OS was defined as the time from study registration to death from any cause. Safety was evaluated as adverse events (AEs) according to the National Cancer Institute Common Terminology Criteria for Adverse Events (CTCAE) version 4.03.

### Statistical analysis

The planned enrollment for this study was at least 70 patients. The sample size of 70 would have provided an estimated PFS of 5 months, 80% power, with a two-sided type I and type II error of 0.05.

Clinicopathologic characteristics was summarized in descriptive statistics. PFS and OS were estimated by the Kaplan-Meier method and the hazard ratios (HRs) and corresponding 95% confidence intervals (CIs) were estimated using the Cox proportional harzard model. Exploratory analyses were performed with the log- rank test. Cox multivariate models were performed based on the univariate analyses results. CBR and ORR were calculated with their 95% CI. Toxicities were summarized, and the maximum grade per patient was used as the summary measure. *P* value less than 0.05 was considered statistically significant. All statistical analyses were managed using SPSS version 23.0.

## Results

### Patients

**Fig. 1:**
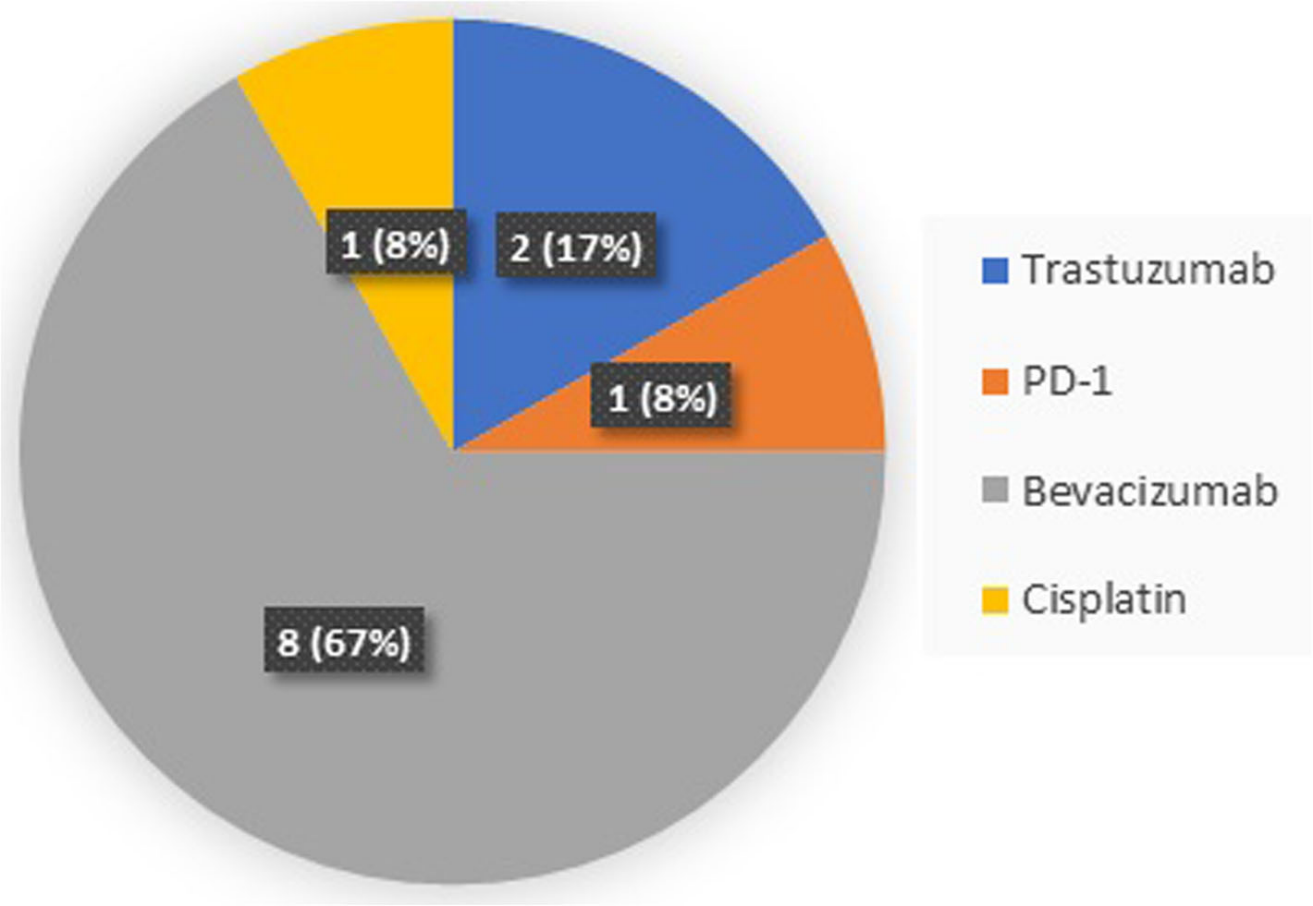
Combined treatment regimens

From March 2016 through September 2020, 80 patients were enrolled on this study and treated at Fudan University Shanghai Cancer Center. All patients received at least one dose of the study treatment and were considered evaluable for toxicity and response. The mean age of patients was 52 years (range 26–86). A majority of patients were estrogen receptor (ER)/progesterone receptor (PR) positive (76.3%), while 22.5% patients were triple negative and 2 patients (2.5%) were human epidermal growth factor 2 (HER2) positive. Most patients were in stable status (ECOG 0–1). Liver metastasis accounts for 67.5% patients, followed by lung metastasis (61.3%) and brain metastasis (6.3%). Patients usually had 3 or more metastasis sites (62.5%). The median prior metastatic chemotherapy regimens were 2 lines. A small minority of patients received combined treatment together with nab-paclitaxel (15%), mostly for targeting therapy, which was shown in Fig. 1. Baseline patient characteristics are summarized in Table 1.

**Table 1:** Patient characteristics at baseline

Characteristics	Total ***N*** = 80	Percentage %
Median Age (range)	52 (26–86)	
ER/PR/HER2 status		
ER and/or PR positive	61	76.3
ER and PR negative	19	23.7
HER2 positive	2	2.5
HER2 negative	78	97.5
Triple negative	18	22.5
ECOG		
0–1	77	96.2
≥ 2	3	3.8
Menopausal status		
Premenopausal	22	27.5
Postmenopausal	58	72.5
Metastatic sites		
Lung	49	61.3
Liver	54	67.5
Brain	5	6.3
Number of metastatic sites		
1–2	30	37.5
≥ 3	50	62.5
Prior metastatic chemotherapy regimens	Median: 2 (Range 0–8)	
0	30	37.5
1	22	27.5
≥ 2	28	35
Combined Treatment		
Yes	12	15
No	68	85

### Efficacy

**Fig. 2:**
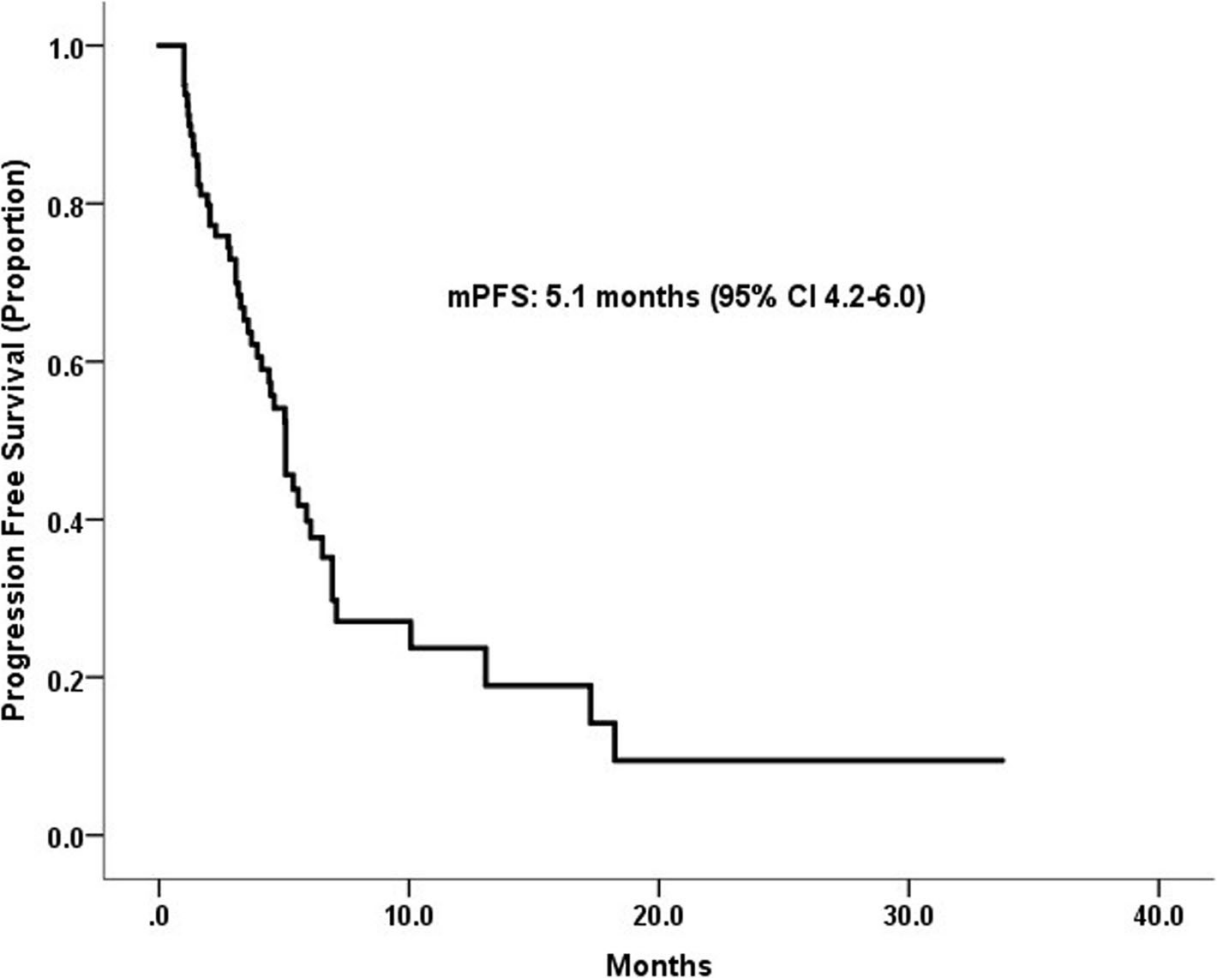
Kaplan–Meier curves for progression-free survival

The median PFS was 5.1 months (95% CI: 4.2–6.0 months, Fig. 2). As for the best treatment response, there were 26(32.5%) patients with PR, 27(33.8%) patients with SD and 27(33.8%) patients with PD, yielding an ORR of 33.8% (95% CI 21.3–43.8%) and CBR of 66.2% (95% CI 56.3–75.0%). Table 2 summarized the outcomes of patients treated on this protocol. The median OS were not reached at the time of analysis.

**Table 2:** Summary of best overall patient response

Response	***N*** = 80 (%)	95% CI
Complete response	0 (0)	
Partial response	26 (32.5)	
Stable disease	27 (33.8)	
Progressive disease	27 (33.8)	
ORR%	33.8	21.3–43.8
CBR%	66.2	56.3–75.0

**Fig. 3:**
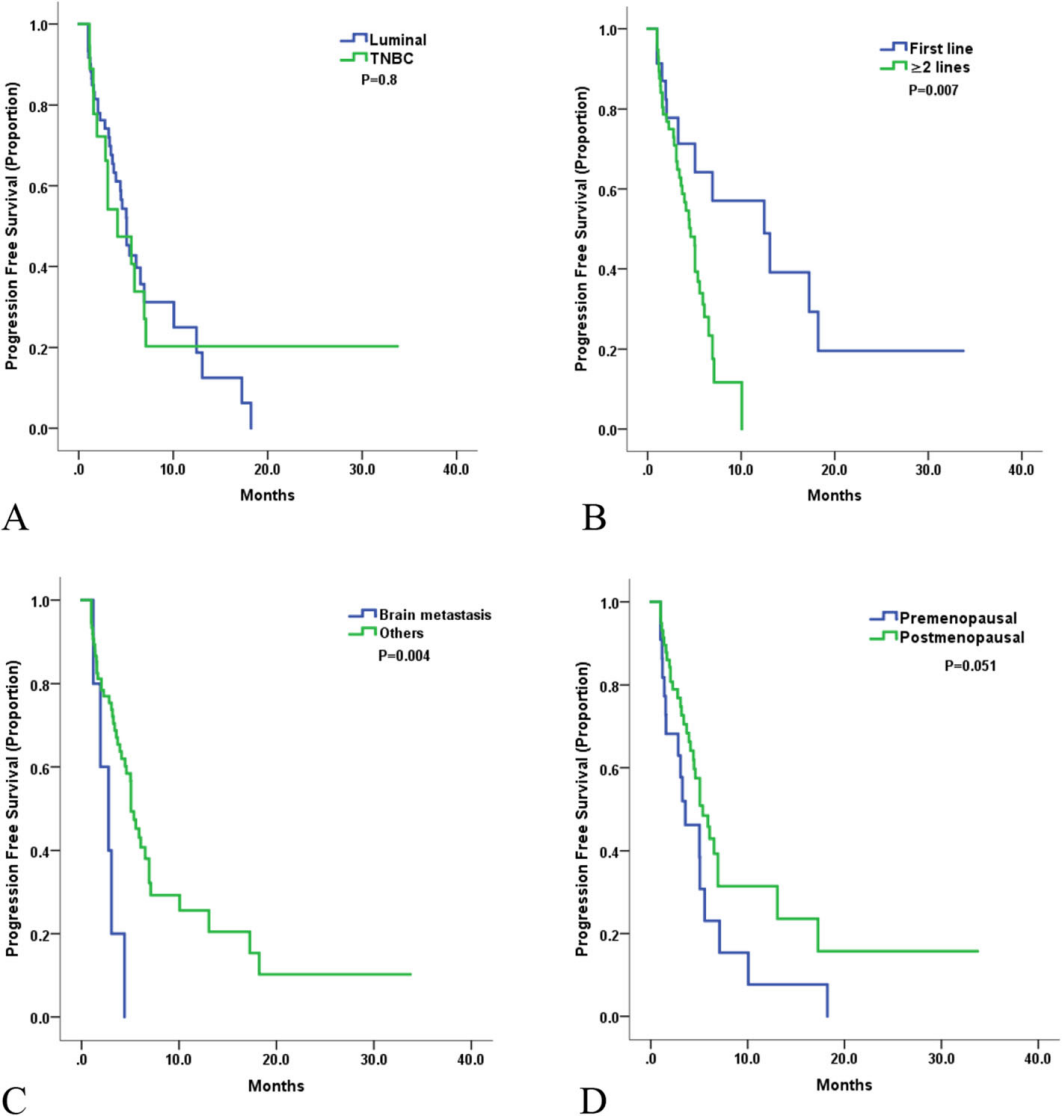
Kaplan–Meier curves for progression-free survival by: A. Molecular types B. Treatment lines C. Brain metastasis or not D. Menopausal status

In subset analysis, the median PFS according to molecular types was 5.1 (95% CI 4.2–6.0) months for luminal and 4.1 months (95% CI 1.5–6.7) for TNBC (*p* = 0.8, Fig. 3A). The median PFS of HER2+ patients was not available due to sample size. Patients receiving nab-paclitaxel as first line had longer PFS compared to later lines [mPFS 12.5 months (95% CI:3.8–21.1) versus 4.6 months (95% CI:3.7–5.5), *P* = 0.007, Fig. 3B]. Patients with brain metastasis showed poorer outcome compared to others [2.8 months (95% CI:1.0–4.6) versus 5.1 months (95% CI: 4.3–5.9), *P* = 0.004, Fig. 3C]. Postmenopausal women had a trend of longer PFS (5.4 months, 95% CI 3.9–6.8) in comparison with premenopausal women (3.6 months, 95% CI 1.3–5.8, *P* = 0.051, Fig. 3D). No significant difference or trend of PFS was observed in patients receiving combined treatment or not; age over 65 or not; had over 3 metastatic sites or not (*P* > 0.1).

Multivariate analysis demonstrated non brain metastasis (adjusted HR 0.31, 95% CI 0.12–0.83, *P* = 0.019) and first line treatment (adjusted HR 0.37, 95% CI 0.17–0.81, *P* = 0.013) as independent predictors of longer PFS.

### Safety

The most common treatment-related, grade ≥ 2 toxicities were neutropenia (42.5%), sensory neuropathy (18.8%), fatigue (6.2%), leukopenia (6.2%), arthralgia/myalgia (5%) and diarrhea (5%). Lung infection was seen in 2 patients (2.5%), which might be owing to neutropenia. Skin rash occurred in 2 patients (2.5%) probably because of allergy. Grade 4 hematologic toxicities were observed in 8 patients (10%). Only one patient (1.2%) developed grade 4 sensory neuropathy. Table 3 summarized the grade 2 and greater toxicities observed in this study thought to be possibly, probably or definitely related to the study treatment.

**Table 3:** Summary of grade 2 and greater toxicities

Adverse events (%)	Toxicity grade (***N*** = 80)			
	Grade 2	Grade 3	Grade 4	All
Neutropenia	21 (26.2)	6 (7.5)	7 (8.8)	34 (42.5)
Sensory Neuropathy	12 (15)	2 (2.5)	1 (1.2)	15 (18.8)
Fatigue	5 (6.2)	0	0	5 (6.2)
Arthralgia / Myalgia	4 (5)	0	0	4 (5)
Diarrhea	1 (1.2)	3 (3.8)	0	4 (5)
Leukopenia	4 (5)	0	1 (1.2)	5 (6.2)
Elevated alanine aminotransferase	2 (2.5)	0	0	2 (2.5)
Infection	2 (2.5)	0	0	2 (2.5)
Rash	2 (2.5)	0	0	2 (2.5)

## Discussion

In this prospective, phase II trial, we examined the efficacy and safety of nab-paclitaxel in MBC patients with visceral metastases. To the best of our knowledge, this is the first direct investigation of nab-paclitaxel in MBC patients with visceral dominant metastases.

We reported a mPFS of 5.1 months and ORR of 33.8%, which conformed to previous studies on nab-paclitaxel for different lines of MBC patients (mPFS 5.75 months, ORR 33%), despite of limitation of visceral metastases [10]. Other studies explored nab-paclitaxel as first line treatment demonstrated a mPFS of 7.1–12.9 months, which could be attributed to first line treatment as well as non-selection of visceral metastasis [11, 14]. With regards to novel chemotherapy eribulin, a phase III trial indicated a mPFS of 4.1 months when treating MBC patients pretreated with anthracycline- and taxane-based therapy, which was similar in comparison to our data [15]. As for targeting therapy of luminal type disease, investigators explored the Palbociclib plus endocrine therapy in patients with visceral metastases and showed a mPFS of 9.2 months for patients with prior resistance to endocrine therapy, which was prolonged possibly owing to ER/PR positive disease and chemotherapy naive for metastatic pattern [5]. Furthermore, this study explored the use of 125 mg/m2 nab-paclitaxel among Asian patients. In all, our data showed a pleasant treatment result of nab-paclitaxel for patients with visceral metastases.

In univariate analysis, although luminal type had higher mPFS value, no significant difference was observed between luminal type and TNBC, which could be attributed to limited sample size of TNBC and heavy pre-treatments. Interestingly, we observed a trend of longer PFS in premenopausal patients compared to postmenopausal women, and we consider a relative younger age to be a possible cause. Partidge et al. conducted a prognosis study enrolling 17,575 breast cancer patients and indicated a significant worse treatment outcome of patients less than 40 years old in contrast with elder patients [16]. Therefore, we recommended young patients to receive more aggressive treatment regimens if possible. Statistical difference was not found between combined treatment and monotherapy, mainly because of a limited sample size.

Non brain metastasis and first line treatment appeared to predict greater PFS even after balancing the known factors. We have discussed the superiority of first line therapy before, which was also demonstrated by clinical and observational trials [10, 12]. Paclitaxel has limited effect on brain metastasis probably due to constrained efflux transport mechanisms present at the blood brain barrier, which leads to a sub-therapeutic and non-uniform drug concentrations in brain metastatic tumor [17, 18]. Although nab-paclitaxel showed an increased brain uptake and toxicity against P-glycoprotein expressing cancer cells of a rat model, clinical trials did not report a positive result [19]. Thus, certain treatment of brain metastasis remained controversial.

The toxicity profile indicated a comparable result to previous studies, with most frequent grade 3/4 AE of neutropenia and sensory neuropathy [10, 11]. Although 3 patients reduced treatment dose because of dose limiting toxicities, the overall safety was acceptable considering a cytotoxic drug. Notably, 3 patients developed unusual grade 3 diarrhea, which could be attributed to individual difference and all patients were relieved after use of loperamide.

## Conclusions

In conclusion, this phase II trial documented satisfactory efficacy and safety of nab-paclitaxel in MBC patients with visceral metastases, providing evidence for relative clinical practice. Patients in first line therapy had better treatment outcome than later lines. For patients with premenopausal status or brain metastasis, further alternatives (for example, combined chemotherapy or targeting therapy) might be required. This study also demonstrated the efficacy and safety of 125 mg/m2 nab-paclitaxel among Asia patients.

## Data Availability

The datasets generated and/or analyzed during the current study are not publicly available due to hospital policy but are available from the corresponding author on reasonable request.

## Abbreviations

MBC: Metastatic breast cancer
HER2: Human epidermal growth factor receptor 2
OS: Overall survival
HR: Hazard ratio
CI: Confidence interval
NCCN: National Comprehensive Cancer Network
RECIST: Response Evaluation Criteria in Solid Tumors
DFI: Disease-free interval
CTCAE: Common Terminology Criteria for Adverse Events
SPSS: Statistical Product and Service Solutions

## References

[1] Fitzmaurice C (2015). The global burden of Cancer 2013. JAMA Oncol.

[2] Bray F, Ferlay J, Soerjomataram I, Siegel RL, Torre LA, Jemal A (2018). Global cancer statistics 2018: GLOBOCAN estimates of incidence and mortality worldwide for 36 cancers in 185 countries. CA Cancer J Clin.

[3] Chen W, Zheng R, Baade PD, Zhang S, Zeng H, Bray F, Jemal A, Yu XQ, He J (2016). Cancer statistics in China, 2015. CA Cancer J Clin.

[4] Gobbini E, Ezzalfani M, Dieras V, Bachelot T, Brain E, Debled M, Jacot W, Mouret-Reynier MA, Goncalves A, Dalenc F, Patsouris A, Ferrero JM, Levy C, Lorgis V, Vanlemmens L, Lefeuvre-Plesse C, Mathoulin-Pelissier S, Petit T, Uwer L, Jouannaud C, Leheurteur M, Lacroix-Triki M, Cleaud AL, Robain M, Courtinard C, Cailliot C, Perol D, Delaloge S (2018). Time trends of overall survival among metastatic breast cancer patients in the real-life ESME cohort. Eur J Cancer.

[5] Turner NC, Finn RS, Martin M, Im SA, DeMichele A, Ettl J, Diéras V, Moulder S, Lipatov O, Colleoni M, Cristofanilli M, Lu DR, Mori A, Giorgetti C, Iyer S, Bartlett CH, Gelmon KA (2018). Clinical considerations of the role of palbociclib in the management of advanced breast cancer patients with and without visceral metastases. Ann Oncol.

[6] Lee ES, Jung SY, Kim JY, Kim JJ, Yoo TK, Kim YG, Lee KS, Lee ES, Kim EK, Min JW, Han W, Noh DY, Moon HG (2016). Identifying the potential long-term survivors among breast cancer patients with distant metastasis. Ann Oncol.

[7] Solomayer EF, Diel IJ, Meyberg GC, Gollan C, Bastert G (2000). Metastatic breast cancer: clinical course, prognosis and therapy related to the first site of metastasis. Breast Cancer Res Treat.

[8] Gradishar WJ, Anderson BO, Abraham J, Aft R, Agnese D, Allison KH, et al. Breast Cancer, Version 3.2020, NCCN Clinical Practice Guidelines in Oncology. J Natl Compr Canc Netw. 2020;18(4):452–78. 10.6004/jnccn.2020.0016. 10.6004/jnccn.2020.0016 32259783

[9] Desai N, Trieu V, Yao Z, Louie L, Ci S, Yang A, Tao C, de T, Beals B, Dykes D, Noker P, Yao R, Labao E, Hawkins M, Soon-Shiong P (2006). Increased antitumor activity, intratumor paclitaxel concentrations, and endothelial cell transport of cremophor-free, albumin-bound paclitaxel, ABI-007, compared with cremophor-based paclitaxel. Clin Cancer Res.

[10] Gradishar WJ, Tjulandin S, Davidson N, Shaw H, Desai N, Bhar P, Hawkins M, O'Shaughnessy J (2005). Phase III trial of nanoparticle albumin-bound paclitaxel compared with polyethylated castor oil-based paclitaxel in women with breast cancer. J Clin Oncol.

[11] Gradishar WJ, Krasnojon D, Cheporov S, Makhson AN, Manikhas GM, Clawson A, Bhar P (2009). Significantly longer progression-free survival with nab-paclitaxel compared with docetaxel as first-line therapy for metastatic breast cancer. J Clin Oncol.

[12] Liang C, Li L, Fraser CD, Ko A, Corzo D, Enger C, Patt D (2015). The treatment patterns, efficacy, and safety of nab ((R))-paclitaxel for the treatment of metastatic breast cancer in the United States: results from health insurance claims analysis. BMC Cancer.

[13] Koumarianou A, Makrantonakis P, Zagouri F, Papadimitriou C, Christopoulou A, Samantas E, Christodoulou C, Psyrri A, Bafaloukos D, Aravantinos G, Papakotoulas P, Baka S, Andreadis C, Alexopoulos A, Bompolaki I, Kampoli Κ, Liori S, Karvounis K, Ardavanis A (2020). ABREAST: a prospective, real-world study on the effect of nab-paclitaxel treatment on clinical outcomes and quality of life of patients with metastatic breast cancer. Breast Cancer Res Treat.

[14] Robert NJ, Diéras V, Glaspy J, Brufsky AM, Bondarenko I, Lipatov ON, Perez EA, Yardley DA, Chan SYT, Zhou X, Phan SC, O'Shaughnessy J (2011). RIBBON-1: randomized, double-blind, placebo-controlled, phase III trial of chemotherapy with or without bevacizumab for first-line treatment of human epidermal growth factor receptor 2-negative, locally recurrent or metastatic breast cancer. J Clin Oncol.

[15] Kaufman PA, Awada A, Twelves C, Yelle L, Perez EA, Velikova G, Olivo MS, He Y, Dutcus CE, Cortes J (2015). Phase III open-label randomized study of eribulin mesylate versus capecitabine in patients with locally advanced or metastatic breast cancer previously treated with an anthracycline and a taxane. J Clin Oncol.

[16] Partridge AH, Hughes ME, Warner ET, Ottesen RA, Wong YN, Edge SB, et al. Subtype-Dependent Relationship Between Young Age at Diagnosis and Breast Cancer Survival. 2016;34(27):3308–14. 10.1200/JCO.2015.65.8013. 10.1200/JCO.2015.65.8013 27480155

[17] Lockman PR, Mittapalli RK, Taskar KS, Rudraraju V, Gril B, Bohn KA, Adkins CE, Roberts A, Thorsheim HR, Gaasch JA, Huang S, Palmieri D, Steeg PS, Smith QR (2010). Heterogeneous blood-tumor barrier permeability determines drug efficacy in experimental brain metastases of breast cancer. Clin Cancer Res.

[18] Loscher W, Potschka H (2005). Role of drug efflux transporters in the brain for drug disposition and treatment of brain diseases. Prog Neurobiol.

[19] Shah N, Mohammad AS, Saralkar P, Sprowls SA, Vickers SD, John D, Tallman RM, Lucke-Wold BP, Jarrell KE, Pinti M, Nolan RL, Lockman PR (2018). Investigational chemotherapy and novel pharmacokinetic mechanisms for the treatment of breast cancer brain metastases. Pharmacol Res.

